# In situ drug release measuring in α-TCP cement by electrochemical impedance spectroscopy

**DOI:** 10.1007/s10856-021-06507-9

**Published:** 2021-04-01

**Authors:** Júnio Augusto Rodrigues Pasqual, Lucas C. Freisleben, Júlio Cesar Colpo, Jose Ramón Jurado Egea, Luis Alberto Loureiro dos Santos, Vânia Caldas de Sousa

**Affiliations:** 1 Department of Materials Engineering, Federal University of Rio Grande do Sul, Porto Alegre, RS Brazil; 2 Brazilian Health Regulatory Agency, Porto Alegre, RS Brazil

## Abstract

The use of drug delivery systems is a good technique to leave the right quantity of medicine in the patient’s body in a suitable dose, because the drug application is delivered directly to the affected region. The current techniques such as HPLC and UV–Vis for the drug delivery calculation has some disadvantages, as the accuracy and the loss of the sample after characterization. With the aim of reducing the amount of material used during the characterization and have a non-destructive test with instantaneous results, the present paper shows the possibility of using electrochemical impedance spectroscopy (EIS) to have a drug delivery measurement during the release phenomena for a calcium phosphate cement (CFC) delivery system with gentamicin sulfate (GS) and lidocaine hydrochloride (LH), at a ratio of 1% and 2%, respectively. The equivalent circuit and the chemical mechanism involved during the measurements have been proposed as a tool to determine the drug delivery profile. The method has been compared with the UV–Vis technique. XRD was realized to verify conditions, before and after release. It was possible to verify the potential for using EIS as an instant technique to quantify drug delivery.

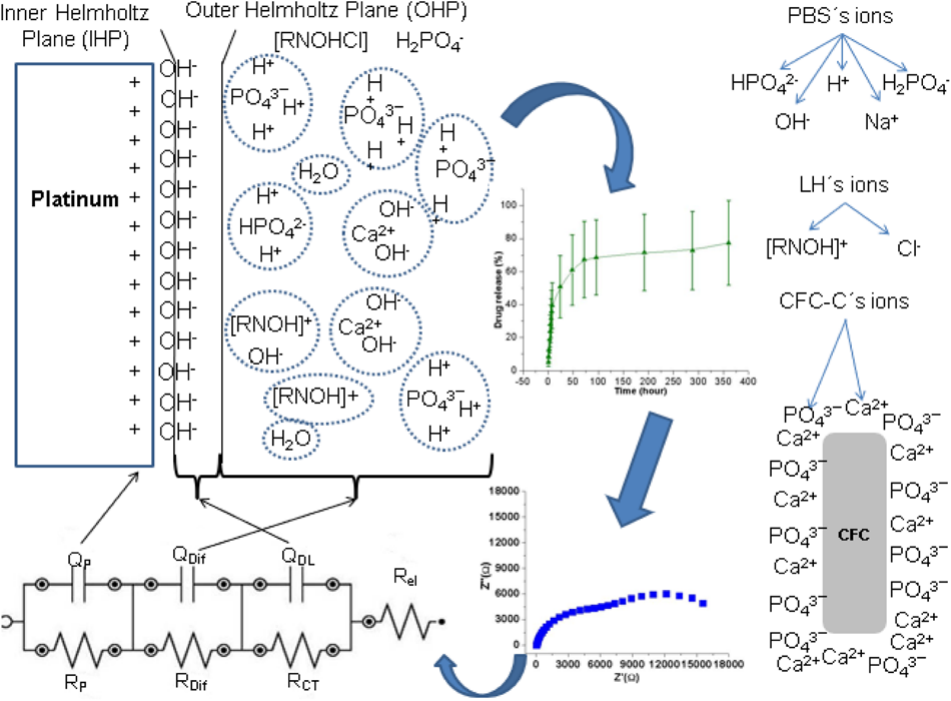

## Introduction

Studies focused on the technological development in the health area have made possible advances that result in tools which increase the range of medical intervention and culminate in the extension of the population life expectancy, year by year. This extension leads to more occurrences of accidents cases caused by bone weakening, common in people of advanced age, or with some diseases that affect this tissue, among other illnesses [1, 2]. The use of materials as temporary or permanent substitutes for human body parts is an important topic which presents itself as an interesting method in the search for improvement in the population’s quality of life. The use of these biomaterials should be in a minimal invasive form, leading to a fast recovery and avoiding side effects, besides seeking maximum benefit for the bones [1, 3]. In the case of ceramic biomaterials, their use is commonly focused on maxillofacial defect treatment, and fracture treatments, among other applications [4, 5].

Over the years, several CFCs, ceramic materials applied as biomaterials, have been studied, including the α-tricalcium phosphate system (α-TCP) which is indicated for forming calcium deficient hydroxyapatite (CDHA) during the setting reaction [4, 6, 7]. Its use as a biomaterial began in the 1990s [1, 4, 8]. The α-TCP cement has calcium and phosphate in proportions similar to that found in the human bone system, which means a greater biocompatibility, i.e., these materials and their products are tolerated by human tissue, and do not cause dysfunctions in the body [6–8]. The α-TCP biocompatibility, porosity and resorbability make this material eligible for use as a carrier for drug charges as it is already widely used with biopolymers [9–12]. The cement degradation velocity is limited by the reaction with the living tissue which has a consequence in drug delivery regulation, allowing an absorption kinetic control of the drug by the body [1, 4]. This control is also due to the cement porosity, the liquid diffusion velocity into the matrix, the crystal’s morphology, the interaction between drug and biomaterial, among other factors [1]. More porous cements have a release velocity with more burst release effect, common in drug delivery systems [2, 4, 9]. The determination of the amount of drug delivered from the cement matrix can be obtained by several well-established methods in drug release studies. These include ultraviolet-visible spectroscopy (UV–Vis) [9, 13–19], High Performance Liquid Chromatography (HPLC) [20–24], and fluorescence polarization immunoassay (FPIA) [25–28].

The kinetics that control the drug release are determined through applied mathematical models, and analysis of the parameters obtained from these models, in order to choose the best model which represents what is the prevailing mechanism during the release [4, 9, 13]. Among the models currently available are the Higuchi, Peppas-Sahlin, and Korsmeyer-Peppas models [4, 9, 13]. These models have some restrictions that limit or prevent their use, depending on the material being studied.

Electrochemical impedance spectroscopy (EIS) is a widely used technique in the characterization of electrical materials that require specific electrical properties, for example, photovoltaic cells, materials submitted to corrosive environments, and fuel cells, amongst others [29–31]. It is a promising technique to determine the amount of drug transported to the body during the release processes, promoting a non-destructive and high sensitivity analysis of the characterized material [32, 33].

The objective of the work presented here is to use EIS, a technique recently used for the measurement of release, as a method to determine the amount of gentamicin sulfate (GS) and lidocaine hydrochloride (LH), at a ratio of 1% and 2%, respectively, released during in vitro testing in a ceramic matrix of an α-TCP phase calcium phosphate cement (CFC). The antibiotic GS, and the local analgesic LH, studied in this work, are widely used in drug delivery systems [4, 15, 16, 20, 34–36].

## Material and methods

### Samples preparation

The CFC samples were obtained by a thermal method using the calcination of dicalcium phosphate dihydrate (DCPD) at 550 °C for 5 h. Then, the gamma-pyrophosphate (γ-CPP) obtained from the DCPD calcination were mixed with calcium carbonate (CaCO_3_) and heated at 1500 °C for 3 h. The material resulting from this last calcination is α-tricalcium phosphate, as shown in Eq. 1. The calcination of the γ-CPP and CaCO_3_ mixture were carried out in alumina crucibles in a low temperature electric furnace SANCHIS (SANCHIS, Brazil) and in a high temperature electric furnace CARBOLITE (CARBOLITE GERO, German and England), respectively. After cooling, the obtained material was de-agglomerated in a 325-mesh sieve, resulting in a powder with 11,78 µm of average size.

1 $${\mathrm{CaCO}}_3 + \gamma - {\mathrm{Ca}}_2{\mathrm{P}}_2{\mathrm{O}}_7 \to \alpha - {\mathrm{Ca}}_3\left( {{\mathrm{PO}}_4} \right)_2\, + \,{\mathrm{CO}}_2$$

A liquid/powder ratio of 0.3 mL/g with 2.5% w/v Na_2_HPO_4_ was used as the accelerator [6, 8]. The cement paste was placed in polypropylene molds of 12 mm (±0.1 mm) in height and 6 mm (±0.1 mm) in diameter and pressed in a uniaxial press, with a load of ~346.8 MPa, for 30 s. Samples with 0.630 g of mean weight were demolded and stored at 37 °C at 100% humidity for 72 h, and then for another 72 h at 37 °C in a dry oven. The mechanical bonding of the particles was guaranteed by the growing of CDHA crystals due to the contact with water from media [8].

**Fig. 1:**
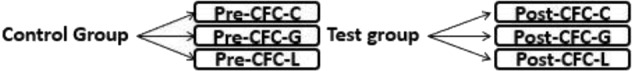
Nomenclature of sample groups studied

Three types of samples were prepared, the first without drug (CFC-C), the second with addition of 1% of GS (CFC-G), and the third with addition of 2% of LH (CFC-L). The samples were divided into those that passed the in vitro release test in phosphate buffer solution (PBS) pH 7.4, called the test group, and those that did not pass the test, called the control group as shown in Fig. 1.

### X-ray diffraction (XRD)

XRD was performed with the purpose of verifying the phases present, observing the influence of drugs and the release test in the production of the phases. The analyses were carried out on the Phillips X’pert MPD diffractometer with a current of 40 mA at 40 kV voltage, a scanning range from 10° to 60° and a step rate of 0.05°. The cards used in the phase identification were 00-009-0348 and 00-029-0359 (α-TCP), 00-009-0169 (β-TCP), and 00-009-0432 (CDHA [Ca_9_(HPO_4_)(PO_4_)_5_OH], obtained from the International Diffraction Data Center.

### Drug release

The in vitro release test was carried out in test tubes with 10 mL of volume. Samples were placed in separate tubes and kept under magnetic stirring (MS) and 37 °C in PBS pH 7.4 for 2 days [11].

To ensure that the entire sample surface area was in contact with the solution, it was suspended to avoid contact with the bottom of the test tube by using of a polythylene raschel mesh.

The tube used for the drug release measurements was adapted for use as an electrochemical cell, especially for that work. Thus, it was possible to monitor the release of the drug in PBS during the test with EIS measurements after the first 8 h, then at 24, 48, and 72 h of test, considering that in many cases, the release phenomena are more intense over the first 8 h [9, 13].

### Electrochemical cell

**Fig. 2:**
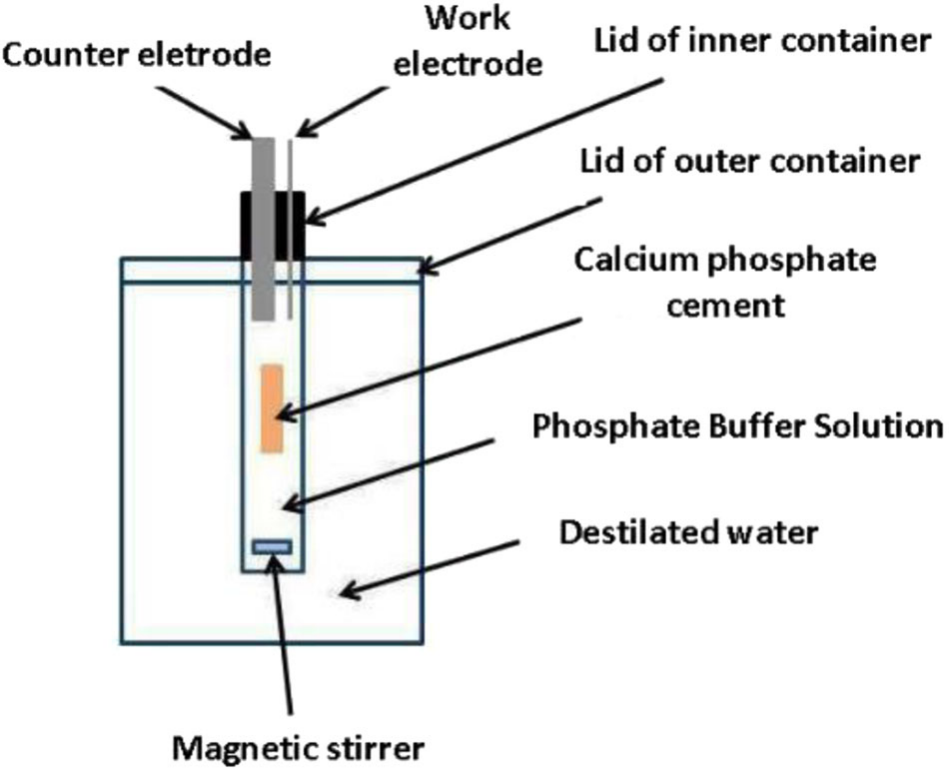
Electrochemical cell for in situ drug delivery

In order to carry out the EIS measurements, it was necessary to develop a specific electrochemical cell to obtain the impedance results during the in vitro drug release test. The glass cell has a cork cover where a platinum plate with dimensions 8 × 34 mm and a thickness of 0.25 mm, defined as a counter electrod, and a wire of the same material with a diameter of 0.25 and 40 mm length, defined as working electrode (WE), were placed. Figure 2 shows the schematic of the assembled electrochemical cell.

The addition of an inert gas during the measurements, and the MS, are two of the means of making the process as homogeneous as possible, reducing interference during the tests [37]. The temperature was controled using a heating plate.

### BIAS variation

The direct current potential (DC potential or BIAS) is entered as an input value in the EIS measurement procedures. The DC potential variation was used in this work as a means to verify the spectrum behavior in order to find the region of greater linearity that, in turn, provides a result with the greater current response applying a smaller potential variation. These measurements were made in potentiostat PGSTAT302N, AUTOLAB (METROHM AUTOLAB, Netherlands).

### Electrochemical impedance spectroscopy (EIS)

The EIS was used in this work as a tool to determine the amount of drug dispersed in PBS pH 7.4 with the passage of time. Measurements in PBS media at 37 °C were performed in the first 8 h, at 24, at 48, and at 72 h of testing. The equipment used for the measurements was the same potentiostat as used in the BIAS measurements.

The DC potential was obtained by BIAS variation and by cyclic voltammetry (CV), carried out in a potential range of 0–2 V, and −2 to 0 V. The frequency range used varied between 10^−1^ and 10^5^ Hz, with an amplitude of 20 mV for all measurements of the studied samples. During in situ impedance measurements, the PBS added at the beginning of the measurement was kept until the end of the experiment. After the measurements were made, NOVA 2.1 software was used to perform fitting of the impedance spectra to obtain the equivalent circuits (ECs) used to represent the phenomena resulting from the interaction between PBS, drugs, CFC, and WE.

The UV–Vis measurements were realized in order to have a commonly used technique to determine the drug delivery profile and compare to the drug delivery profile obtained by EIS. Samples of 10 mL each were collected at 30 min, 1, 2, 3, 4, 5, 6, 7, 8, 24, 48, 72, 96, 192, 288, and 360 h, then stored in a freezer for later thawing and measurement of the amounts of drug released in solution by this technique.

## Results and discussion

### Phases formation influenced by drug presence and release test

**Fig. 3:**
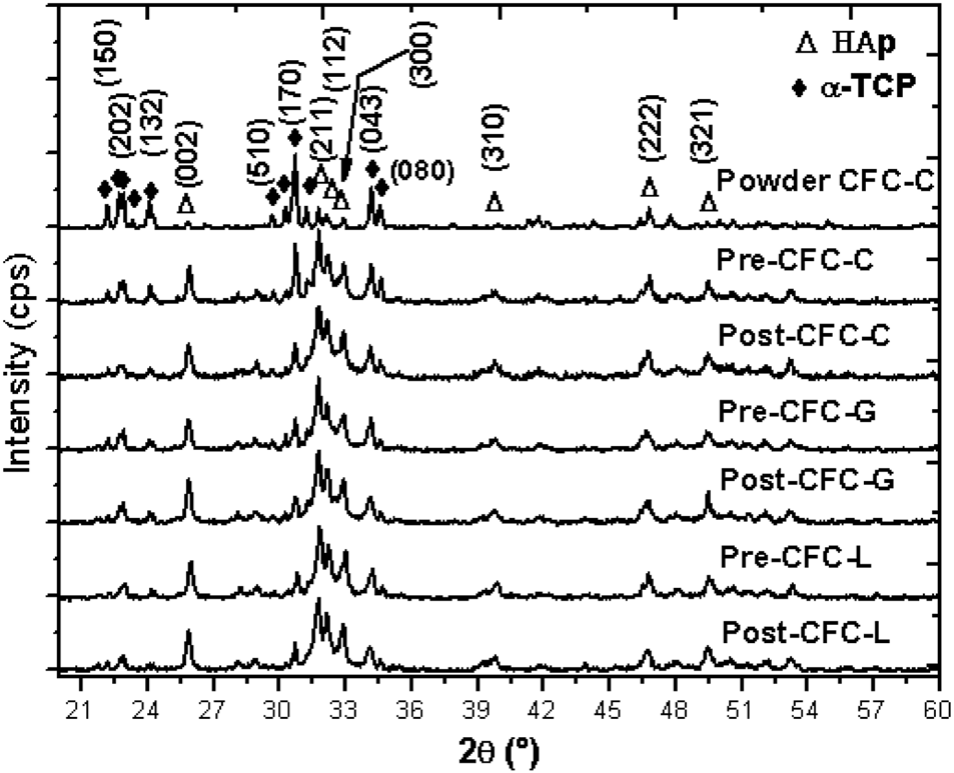
Cement samples diffractograms before and after release test

Figure 3 shows the diffractograms for the CFC samples from the control group (Pre-CFC-C, Pre-CFC-L and Pre-CFC-G), the test group (Post-CFC-C, Post-CFC-L and Post-CFC-G) and the CFC-C powder. It is possible to verify, from this figure that there were no significant changes in the studied materials which presented, as predominant phases, the α-TCP and the CDHA [38, 39].

It is also possible to conclude that neither the contact with PBS under MS, nor the addition of GS and LH, caused formation of new phases in the ceramic material, as already reported in the literature [13].

The change occurs only in the formation of (002), (310), and (321) planes of CDHA whose are observed on the samples after molding step (CFC-C, CFC-G and CFC-L), that is as a consequence of humidity contact, before and after EIS measurements. PBS medium did not have significance effects on the presence of planes. The planes of CDHA formed shown an increasing to all the three types of samples and also it is possible to see that these peaks are more narrow and sharp suggesting an increase of crystallinity.

### BIAS selection by CV and potential DC variation

In order to establish the better BIAS to be used to determine the drug release, potential DC variation by EIS and CV was used.

**Fig. 4:**
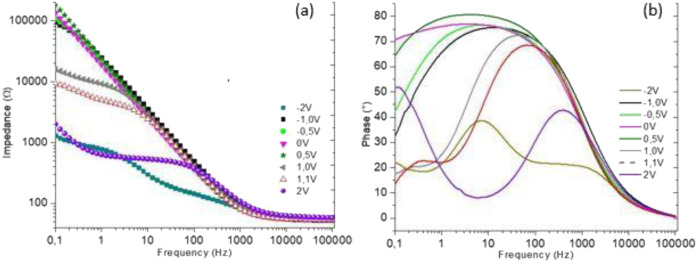
Different BIAS values influencing the impedance results (**a**) and the phase angle (**b**) of PBS solution with a frequency of 10^−1^ to 10^5^ Hz in an environment with MS

The spectra obtained by EIS, shown in the Bode diagrams in Fig. 4, are PBS: without CFC, without drug, and in an environment without MS, for different values of BIAS with a frequency range of 10^−1^ to 10^5^ Hz. They present variations in their formats as changes occurred during the applied BIAS. From the negative potentials, it is possible to observe changes around 100 KΩ in the impedance values for potential intervals close to zero (between −1 V and 0.5 V). It is also possible to observe that potential values above 0.5 V show an order of magnitude of the impedance results of around 10^4^ Ω or below.

**Fig. 5:**
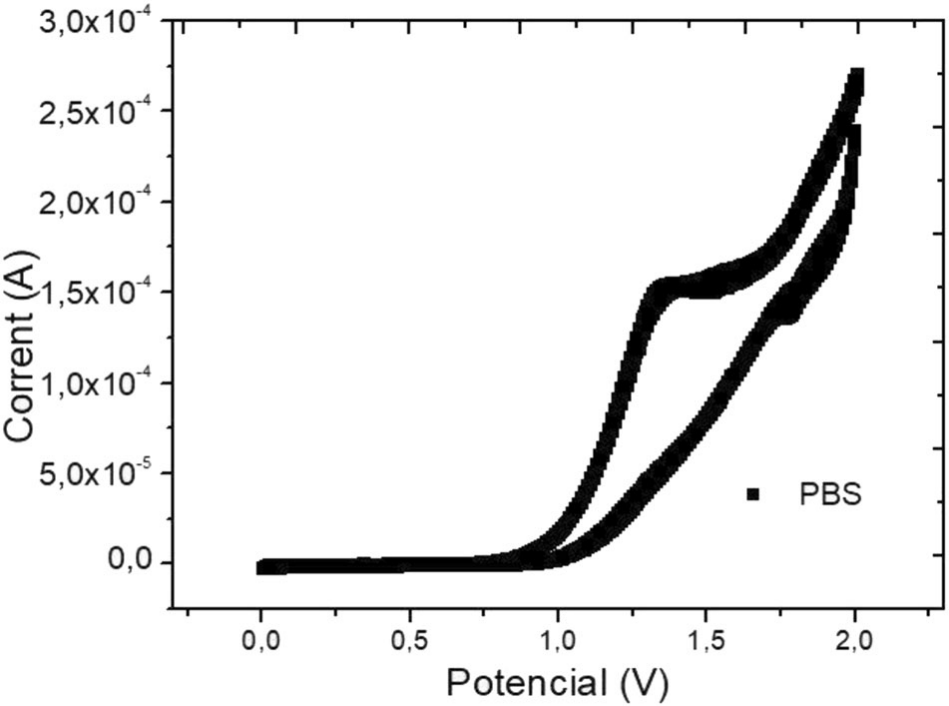
PBS pH 7.4 voltammogram, 0–2 V, and without MS

It is believed that the high resistance values in some potentials are linked to the fact that these potentials are not in the linear region, culminating in a low current response even with high potential application. The reduction of resistance values occurs close to the range in which the linearity region is present. This value is around 1.1 V. The information is confirmed by the PBS voltammogram without the presence of drug or CFC in a non-MS environment, as shown in Fig. 5, which was obtained after a 30-cycle CV measurement.

This resistance variation is linked to the polarization process at the electrode/electrolyte interface, the closer to the linearity region, the lower the polarization resistance [11].

### Analyses of drug and cement by EIS and UV–Vis

The EIS studies were performed in order to verify the correlation between drug concentration and variation in the impedance results. Several tests had as their objective the standardization of a method, in view of noise elimination or reduction, that could influence the obtained results. The presence of these interferences can provoke a masked measure response, influenced by phenomena other than those related to the presence of CFC and drugs in PBS.

**Fig. 6:**
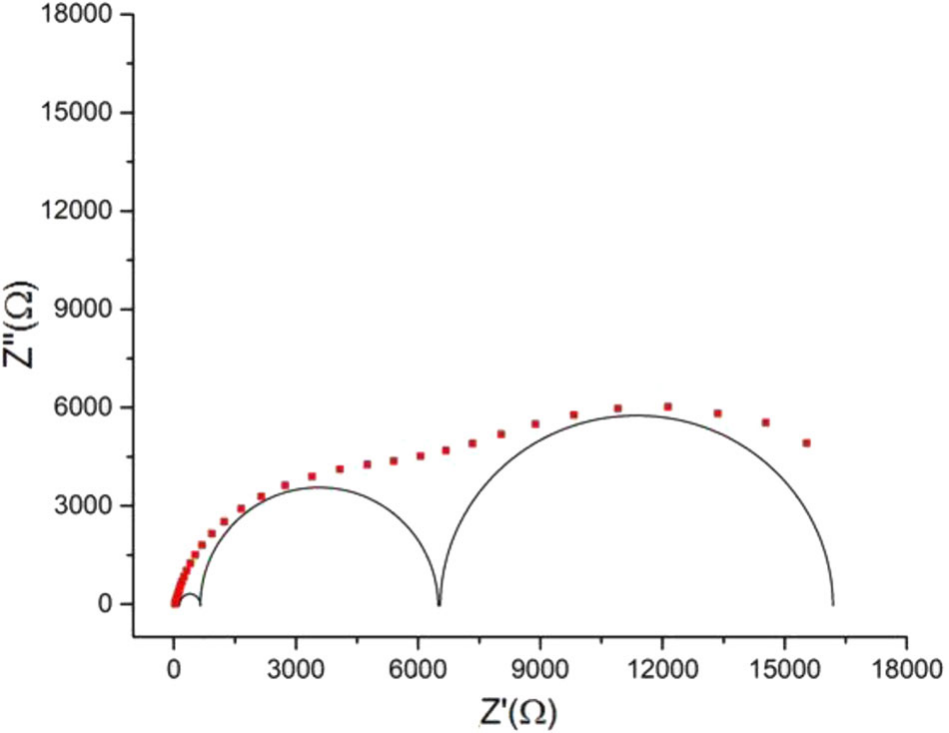
Pattern spectrum for impedance measurements of PBS, CFC-C, CFC-G, and CFC-G

The fitting made it possible to verify that in the analysis of PBS or CFC-C, CFC-G, and CFC-L in PBS, the same EC represents the phenomena at the electrode/electrolyte interface. In all cases, the spectra presented the pattern shown in Fig. 6 below.

Systems changing as a consequence of the increased presence of the ions from PBS, CFC, and drugs, influenced the values of the circuit elements obtained, post fitting, in each sample. It was concluded that the presence of these ions has an effect on the system impedance without provoking new electrochemical phenomena beyond those already expected due to the interaction between the PBS ions and the platinum electrode.

**Fig. 7:**
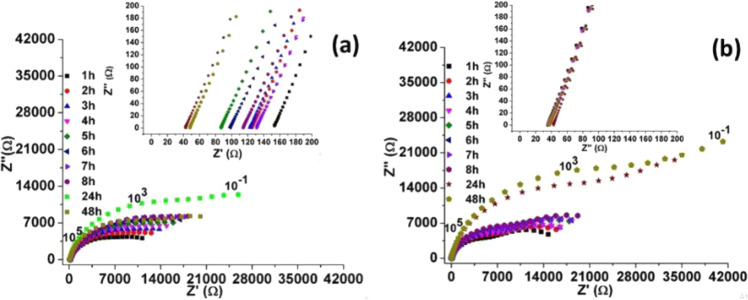
EIS measurements with 1.1 V BIAS for PBS with CFC without drug presence in environment without stirring (**a**) and with stirring (**b**)

The MS was used during the measurements to ensure that ions were always moving within the electrochemical cell during the release test, thus avoiding species decantation over time. Figure 7 shows the effect of the MS presence on the medium over time, during potential impedance measurements of 1.1 V and with a frequency range of 10^−1^ to 10^5^ Hz.

Measurements at 48 h show a significant reduction in the resistance results in Fig. 7a. However, in the environment with MS (Fig. 7b), this behavior disappears in the results of the 48 h test, confirming the ions decantation effect, over time, in environments without MS and so justifying the need for MS during the measurements to obtain results linked to the release process. In addition, it is possible to verify an increase in the resistance values as a consequence of the movement of the species in solution due to the influence of the AM. The impedance averages with MS went from 1.85 to 3.12 KΩ.

**Fig. 8:**
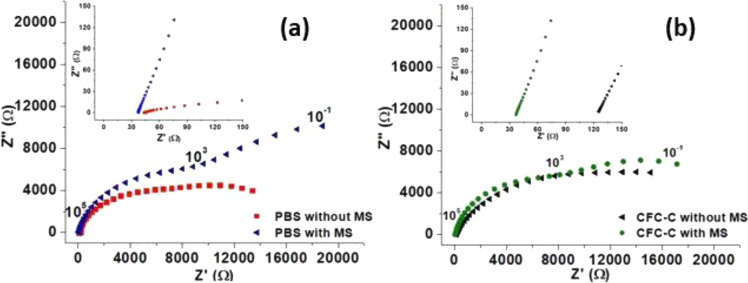
First 8 h averages of EIS hourly measurements for PBS (Fig. 8a) with and without CFC-C (Fig. 8b) in environment with and without stirring

Figure 8 exhibits the Nyquist diagram of PBS pH 7.4 in four situations, all of them with a potential application of 1.1 V and a frequency of 10^−1^ to 10^5^ Hz. The values in these diagrams are averages of the first 8 hourly resistance values of three different measurements. Comparing the results, it is possible to verify that in the resistance values for the result of PBS without MS and without CFC-C (Fig. 8a), the resistance is lower in relation to the result for PBS with MS, with an average increase of 28.66% after MS. These results show the influence of the magnetic field over the species in solution, possibly because of the species drag increasing the accumulation of these ions in the interface between the WE and the electrolyte (PBS), besides the effect on the species decantation as already mentioned. The same effect occurs on the samples of Fig. 8b (with CFC-C). Impedance values undergo an average increase of approximately 13.62% after MS.

Also, in Fig. 8, the comparison between the PBS (Fig. 8a) with CFC-C (Fig. 8b) results in an environment with and without MS allows the verification of how the cement presence has a significant effect on the impedance values, especially when MS occurs where the CFC-C presence caused a reduction in impedance values of around 10–15%. Thus, it was possible to conclude that, when in MS, the PO_4_^3−^ and Ca^2+^ ions lead to system conductivity growth when dispersed in the electrolyte.

**Fig. 9:**
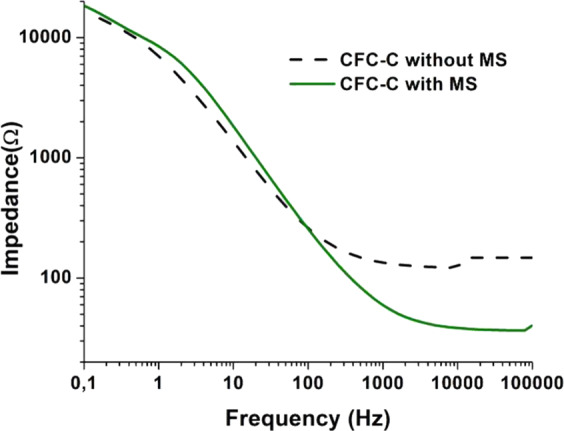
Impedance variation in environment with and without MS for CFC-C

It is also possible to conclude that, in the case where CFC is immersed in PBS (Fig. 9), the ion movement caused by MS results in an increase in impedance values, especially at low frequencies. Figure 9 shows the change of these values with frequency variation, applied during the impedance measurements. The graph analysis shows an impedance value increase due to MS at low frequencies, and an inversion of this behavior as the frequency values increase. Thus, it is possible to connect common electrochemical phenomena at low frequencies, such as charge transfer and electrical double layer, being influenced by the MS in order to have their resistance values increased due to the difficulty of their happening as a consequence of the greater species movement in the electrolyte. On the other hand, the high frequencies phenomena, like electrode polarization, are favored by MS as can be seen in the impedance values shown in the two graphs [40].

**Fig. 10:**
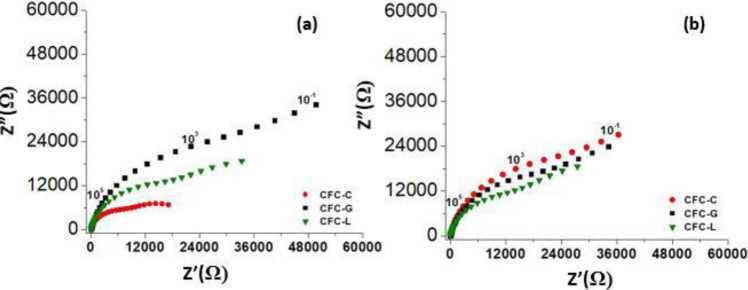
Nyquist diagrams for EIS measurements in CFC-C, CFC-G, and CFC-L, in PBS pH 7.4 after two days (Fig. 10a) and after 30 days (Fig. 10b)

In Fig. 10, it is possible to see the Nyquist diagrams for the three types of CFC samples studied after two days (Fig. 10a) and after 30 days (Fig. 10b) of release test. Observing this figure, it is possible to verify the increase in resistance values with the addition of the two types of drug. The GS addition caused an increase of ~73.33% in impedance values, whereas the LH presence caused an increase of ~46.01% in these values, compared to the results for CFC-C. This increase can be attributed to the interaction between drug ions and the ions present in the solution (PO_4_^3−^, Ca^2+^, H_2_PO_4_^−^, HPO_4_^2−^, Na^+^ and OH^−^), phenomena already discussed in previous studies [6, 41].

The graph to the right of Fig. 10b shows the 8 h measurements average with the samples that were tested for 30 days and then placed in contact with new PBS to perform the impedance measurements that are presented in that figure. Due to contact with the solution during all the 30 days release test, and afterward during these new measurements (2 more days), the cement continued to react with the aqueous environment and to form CDHA, as expected for α-TCP cement. Since the GS amount is lower due to the large part release during the 30 days test, there was a reduction in the CFC-G sample resistance, comparing Fig. 10a to Fig. 10b. In the case of the CFC-L sample, the resistance values appear to remain at the same level when comparing the measurements of this sample in Fig. 10a, b. For the CFC-C sample, there was an increase in the resistance value in the measurements after 30 days of release.

It is possible that even with the reduction of the drug presence in solution, in the case of CFC-L, the impedance remained at the same level because, along with this effect leading to the reduction of resistance, the growth of CDHA crystals occurs. These crystals contribute to increase the resistance of the region, keeping this value close to the previous one in the final balance of each influence.

In the case of the CFC-G sample, the resistance value reduction occurs because the GS ions have more effect on the system resistance than the Ca^2+^ and PO_4_^3−^ ions. In view of this effect, the reduction of the amount of GS ions interferes more in the result than the same reduction for the cement ions.

Finally, in the case of the CFC-C sample, the resistance is increased purely by the presence of cement ions in solution which, in turn, increases due to the greater presence of CDHA crystals.

The authors believe that the difference in resistance values for the three types of samples is also linked to the presence of species in solution with a larger chain length, due to the dissolution of GS ions, for CFC-G, and LH samples for CFC-L samples, hampering the species mobility in PBS, so increasing the system resistance. The size of the chains of each drug seems to be directly related to the mobility of the system. The GS (C_60_H_125_N_15_O_25_S) is a salt with higher chains when compared to LH (C_14_H_23_ClN_2_O), and even more when compared to CFC-C, showing a relation between chain size and medium resistivity as can be observed in the comparison between the presented results in Fig. 10.

In addition to having a larger chain, GS also has a higher degree of dissolution compared to LH. In the case of GS, the dissolution is more complex, with more species being formed and with a dissociation percentage, at pH 7.4, above 90%. For LH, the dissociation occurs in ~70% of the material at pH 7.4, providing less dispersed ions and so having a lesser influence than the GS in the species movement in PBS.

**Fig. 11:**
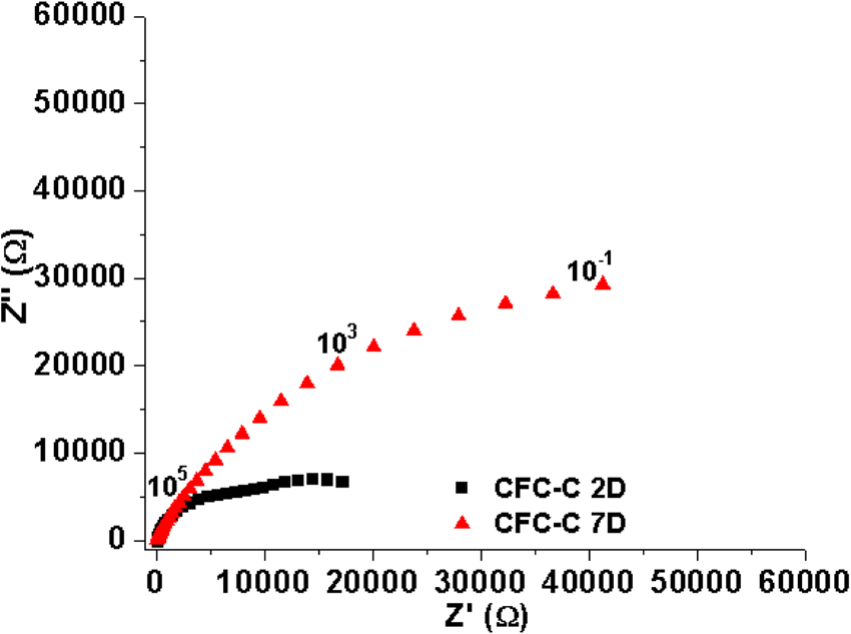
Nyquist diagram CFC-C samples

The correlation between the resistance values variation and the presence of CDHA crystals was based on the results that follow in Fig. 11 below. They are a comparison of the impedance results between two samples. One was kept at 100% humidity for 2 days and the other one was placed in the same environment for 7 days. The higher contact time with the humidity of the 7 day sample resulted in a higher growth of CDHA crystals, which culminated in a higher resistance value for the sample CFC-C 7D, as can be seen in the figure in question. The larger crystals allow a greater contact area between liquid and CFC, culminating in a greater release of PO_4_^3−^ and Ca^2+^.

Although there is a greater species presence in solution due to the presence of drug ions dispersed in PBS, in addition to CFC ions and ions from PBS itself, there is no reduction of total resistance over time for most of the studied samples. The exception is a specific event for the result of the CFC in PBS with no drug and no MS (Fig. 7a), which can be justified by ion decantation over time.

**Fig. 12:**
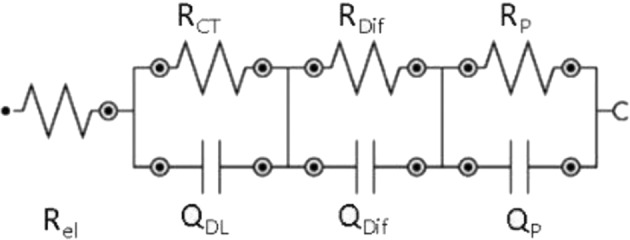
Equivalent circuit for EIS measurements of CFC-C, CFC-G, and CFC-L

The EC shown in Fig. 12 is related to the impedance results of samples CFC-C, CFC-G, and CFC-L. There is a change in the EC values due to the drugs presence in solution, but this presence does not instigate the occurrence of new electrochemical phenomena beyond those expected for the CFC-C samples.

The EC is composed of four resistors and three pseudo capacitors. The first resistance of the EC is considered as the electrolyte resistance (*R*_el_) and presents stability because there are no discrepant values (more than one decimal place) between each measurement. In the first 8 h, this resistance presented 45.97 Ω as an average value with a minimum value of 45.08 Ω and a maximum value of 47.18 Ω. During the 24 and 48 h measurements, the electrolyte resistance reduction reached 35 Ω in the last measurement.

The second resistance is attributed to the electrical charge transfer (*R*_CT_) during the measurements. In Fig. 4 it is possible to verify the reduction in this resistance with the increase of the applied BIAS, which refers to a greater facility of charge transfer following the DC potential variation. Parallel to this resistance is the first pseudo-capacitor that represents the ion accumulation phenomenon on the electrode surface, resulting from the capacitive current generation with the potential difference application in the WE, culminating in the double electric layer (*Q*_DL_) [42, 43].

The third resistance is related to the interaction between CFC ions and electrolyte (*R*_Dif_). The accumulation of these ions near the platinum electrode surface causes the creation of an interface that has its formation difficulty measured by this resistance. Besides this resistance, the capacitance linked to the positioning of these species to form the CFC/PBS interfacial region is given by the pseudo-capacitive circuit element (*Q*_Dif_).

The fourth resistance represents the ion adsorption and diffusion phenomenon through the electrode/electrolyte interface, causing the formation of an ions layer on the WE surface that results in a phenomenon of polarization resistance (*R*_P_) in this region [43]. Finally, the third pseudo-capacitor (*Q*_P_), that appears in parallel with the third resistance, is considered as the polarization effect representation that occurs in the WE, culminating in the adsorption and/or ion diffusion in this region.

**Fig. 13:**
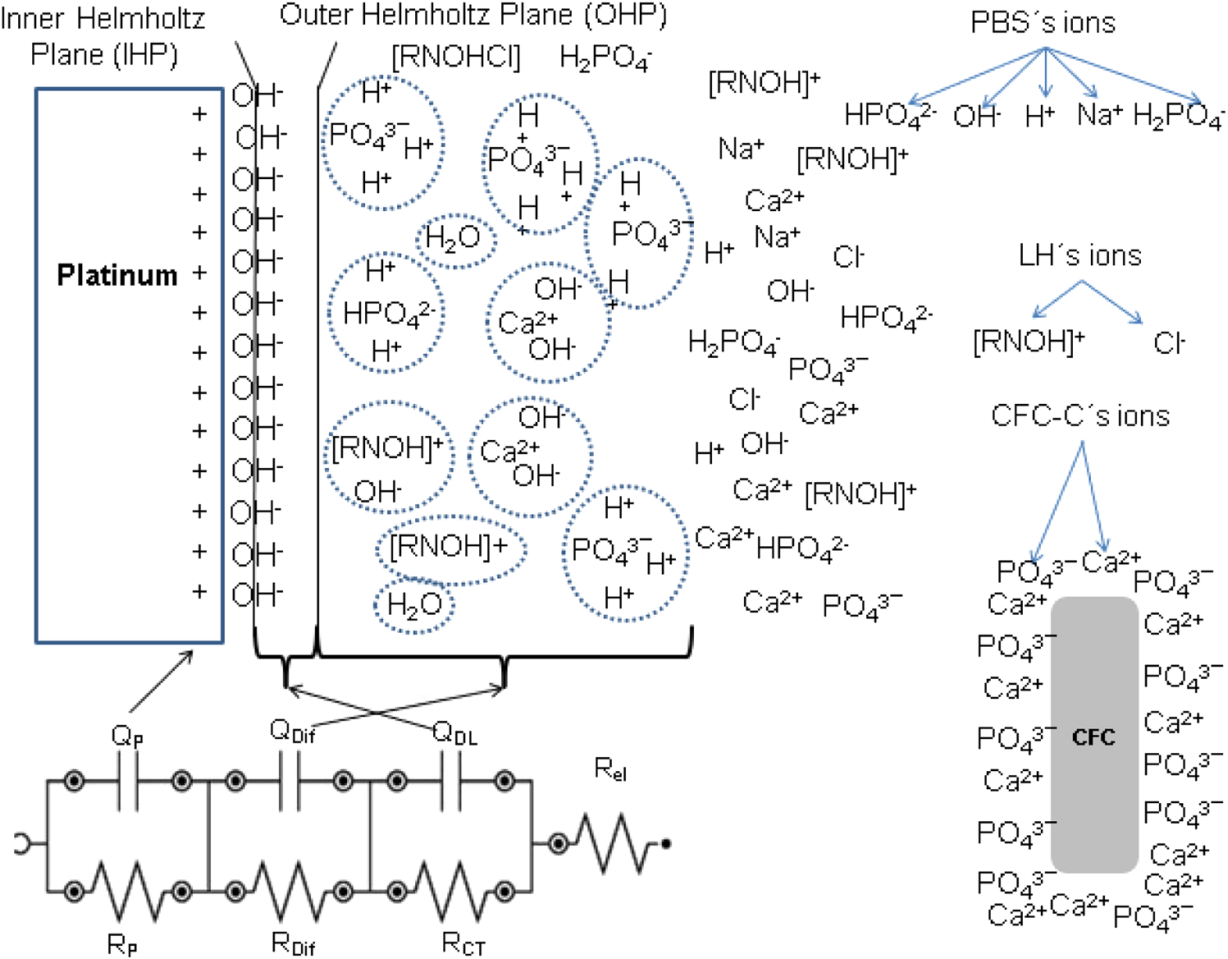
Phenomena schematic during EIS measurement for CFC-L sample

Through Fig. 13, it is possible to correlate EC and the phenomena that occur during CFC-C exposure in PBS pH.7.4 in the electrochemical cell fabricated for this study.

In the results of Fig. 8 it is possible to observe that the CFC presence by itself already causes change in the solution resistance values. The variation of the solution resistance, due the CFC-C presence in this study, was correlated to the increase of the species presence. The PO_4_^3-^ and Ca^2+^ ions alter the species movement, varying the conductivity of the medium.

In the case of CFC-G and CFC-L samples, there is the same EC as shown in Fig. 12. For these types of samples, a significant increase occurred in the circuit element values that compose the EC, an increase that was credited to the interaction between drug ions and other species already present in the electrolyte. In the study of the phenomena that occur for the three types of samples (CFC-C, CFC-G, and CFC-L), the interactions that occur are shown in Fig. 13, which presents the scheme that represents the phenomena resulting from the interaction between CL and solution, but it is also available for CFC-G and CFC-C.

Each circuit element shown in Fig. 12 responds in a different way for each sample because their phenomena are influenced at different intensities by the presence of each drug.

**Fig. 14:**
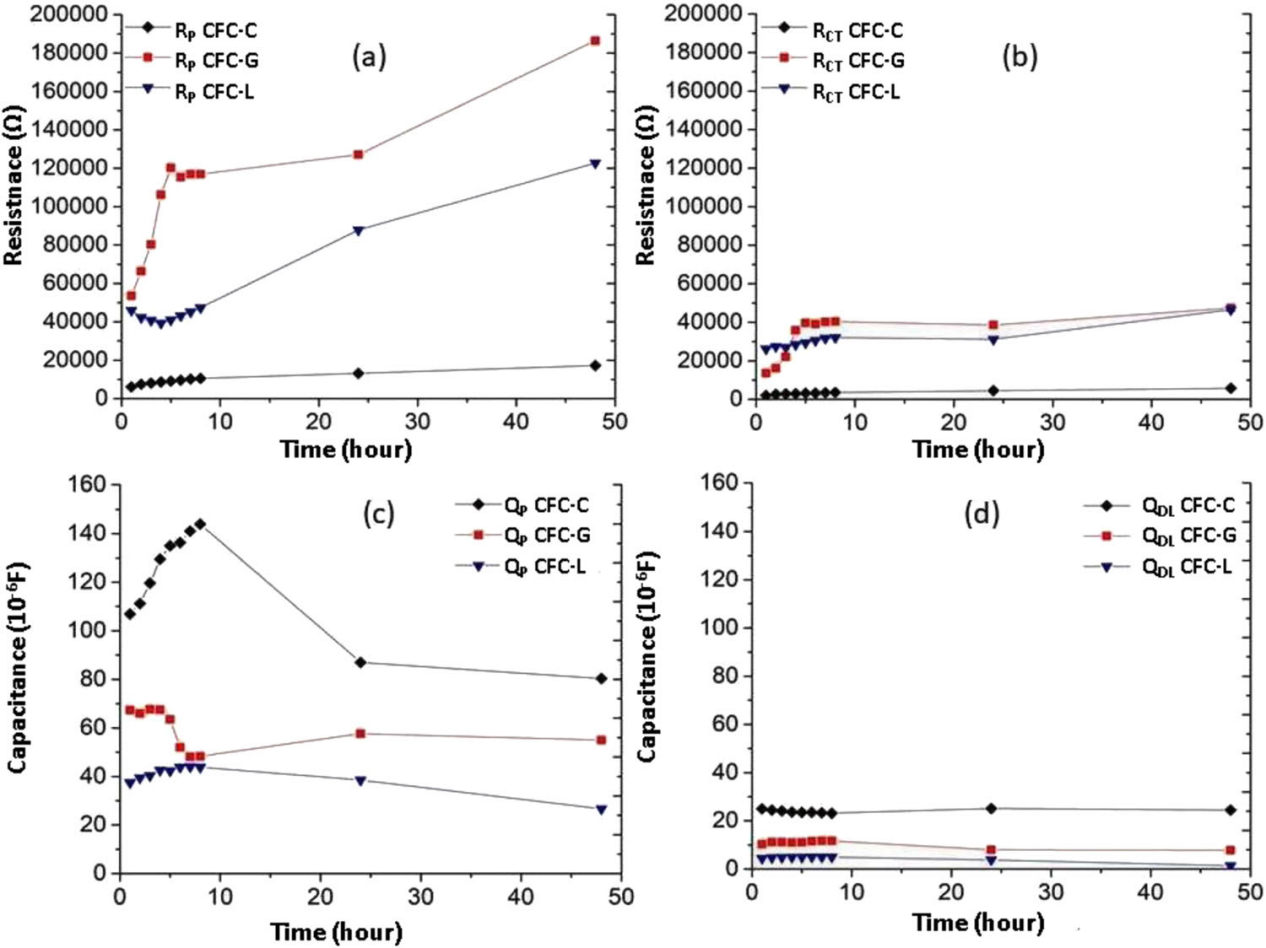
Resistances *R*_P_ (**a**) and *R*_Dif_ (**b**) and capacitances *Q*_P_ (**c**) and *Q*_Dif_ (**d**) for EIS measurements to CFC-C, CFC-G, and CFC-L samples

The Fig. 14 shows the resistances *R*_P_ (Fig. 14a) and _RCT_ (Fig. 14b) and the capacitances *Q*_P_ (Fig. 14c) and *Q*_DL_ (Fig. 14d) obtained after fitting of the results for the three types of samples (CFC-C, CFC-G, and CFC-L), which vary according to the increase in drug concentration of the solution. The samples showed no significant variation for *R*_CT_ values (less than 100 Ω) and because of its discrepancy, compared to the values of *R*_P_ and *R*_Dif_, the authors preferred to not include *R*_CT_ and *Q*_DL_ values on the graphs of Fig. 14.

Through the analysis of the graphs (Fig. 14), it is possible to conclude that *R*_P_ is much more influenced by the increase of the drugs presence in PBS, being more sensitive to the presence of GS in solution than to the presence of LH, as can be observed by the high resistance values for *R*_P_ for these two samples over time (Fig. 14a, b). The lowest values of *R*_P_ and *R*_CT_, as expected, appear in an environment without drug presence. Results related to *R*_P_ (Fig. 14a) and *R*_Dif_ (Fig. 14b) varied according to Table 1 below.

**Table 1:** Variation of *R*_Dif_ and *R*_P_ values due to the presence of GS and LH compared to CFC-C sample

Sample	Increase average (%)	
	*R*_Dif_	*R*_P_
CFC-C	–	–
CFC-G	218.03	632.59
CFC-L	142.72	176.93

This variation is comparing CFC-G and CFC-L to the resistance values for the CFC-C samples. The addition of drugs causes changes of behavior in the electrode vicinity. The effect is more significant for the CFC-G sample, which has the longest molecular chain compared to the CFC-L. Table 2 shows the values of *X*^2^ after fitting of each sample, presenting the convergence between the proposed circuits and the spectra obtained after EIS measurement.

**Table 2:** *X*^2^ for each sample after fitting

Time (hour)	*X*^2^		
	CFC-C	CFC-G	CFC-L
1	0.0032168	0.0060777	0.0017699
2	0.0025071	0.0053064	0.0021358
3	0.0025763	0.0050714	0.0012120
4	0.0027445	0.0039181	0.0022836
5	0.0025148	0.0052006	0.0013600
6	0.0026660	0.0053020	0.0012723
7	0.0027493	0.0054845	0.0019470
8	0.0028766	0.0041331	0.0019479
24	0.0031154	0.0010618	0.0022112
48	0.0032596	0.0004819	0.0038466
Average	0.0028226	0.0042038	0.0019986

Other types of circuits were tested in order to find those that best represent the electrochemical phenomena at the electrode/electrolyte interfaces (R(RQ)(RQ) and R([RW]Q)), but the EC presented in the this study was the one that had the most satisfactory (closest to zero) *X*^2^ values in comparison with the others.

Using the information that *R*_P_ is the circuit element more sensitive to the drug delivery phenomena, it was possible to obtain an equation relating the known concentration and *R*_P_ obtained by fitting. The equations for the CFC-G and CFC-L are presented in Table 3 with the respective dispersion relation between the real values and the equation estimation (*R*^2^).

**Table 3:** Equations and R^2^ obtained from EIS calibration curves for CFC-G and CFC-L

Drug	Equation	*R*^2^
Gentamicin sulfate	*Y* − 0.19067*X*	0.95839
Lidocaine hydrochloride	1.8078*X* − 0.1944	0.96295

**Fig. 15:**
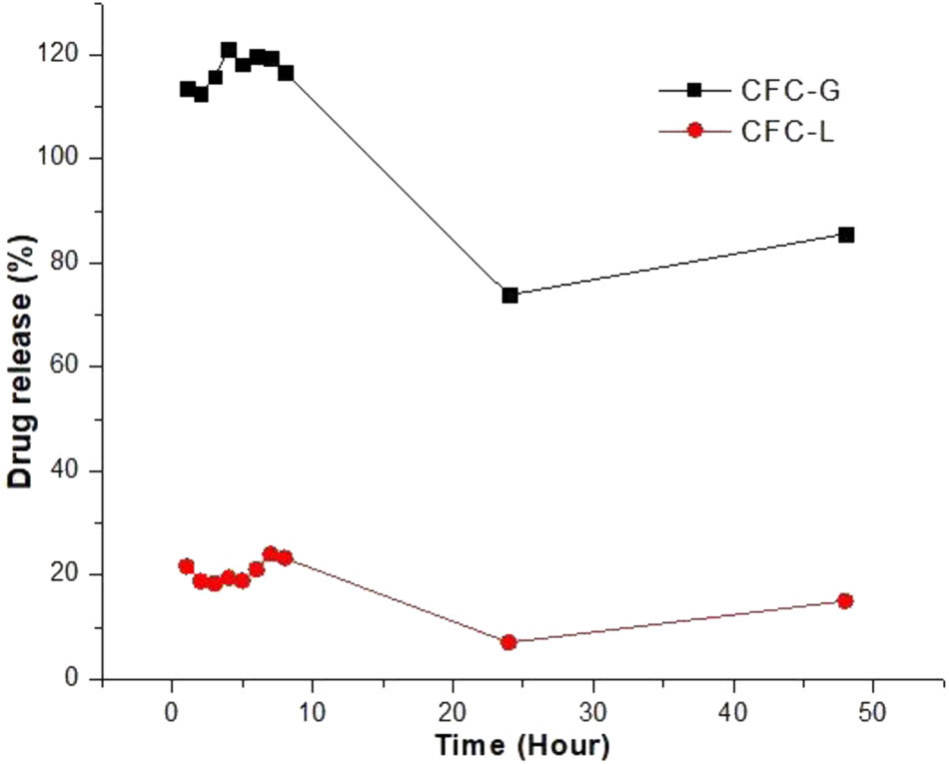
EIS drug release profile for CFC-G and CFC-L samples

The *R*_P_ values for CFC-C samples were subtracted from the *R*_P_ values for CFC-G and for CFC-L, as well as UV–Vis analysis, to remove the CFC-C influences that can interfere on the CFC-C measurements. Figure 15 shows the release profile for the samples studied.

In order to compare the drug release profile, UV–Vis measurements were made on CFC-L and CFC-G samples. The equations obtained of the calibration curve graph after removing the CFC-C influence are shown in Table 4.

**Table 4:** Equations and R^2^ obtained from UV-Vis calibration curves for CFC-G and CFC-L

Drug	Equation	*R*^2^
Gentamicin sulfate	0.07607*x*	0.99983
Lidocaine hydrochloride	1.67384*x*	0.99751

**Fig. 16:**
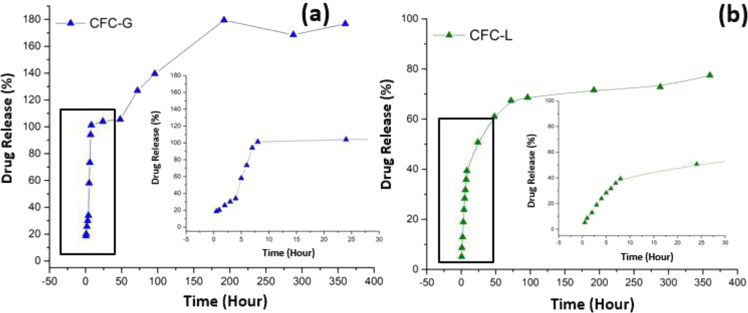
Drug release profiles for **a** CFC-G and **b** CFC-L samples

After to obtain the equations using the calibration curves (concentration versus *R*_P_ course for GS and LH), these made it possible to find the quantities of drugs released into solution over time. Figure 16 shows the profiles obtained after performing triplicate analyses of the sample solutions for 15 days using the UV–Vis technique for GS (Fig. 16a) and for LH (Fig. 16b).

As can be seen, the profile obtained for the EIS results (Fig. 15) does not agree with the values found for the UV–Vis measurement results (Fig. 16). Not even the effect of ion accumulation is observed over time. This observation suggests that subtracting the effects of CFC-C is not an appropriate option to allow the visualization of the *R*_P_ variation, related exclusively to the effect of the presence of the drug in solution. The difficulty of separating the phenomena for each source that makes up the system (PBS, CFC-C, and drug ions) is supported by the UV–Vis results for GS release, where the percentage release values are above 100%, indicating the possibility of interaction between drug ions and other species dispersed in solution.

However, when considering accumulation of the amount of drug released in solution after each time interval (cumulative sum of the amount of drug released after each hour), the profile approximates to the profile found in the UV–Vis results, with an apparent noise reduction inherent in the conventionally used drug release measurement methodology.

**Fig. 17:**
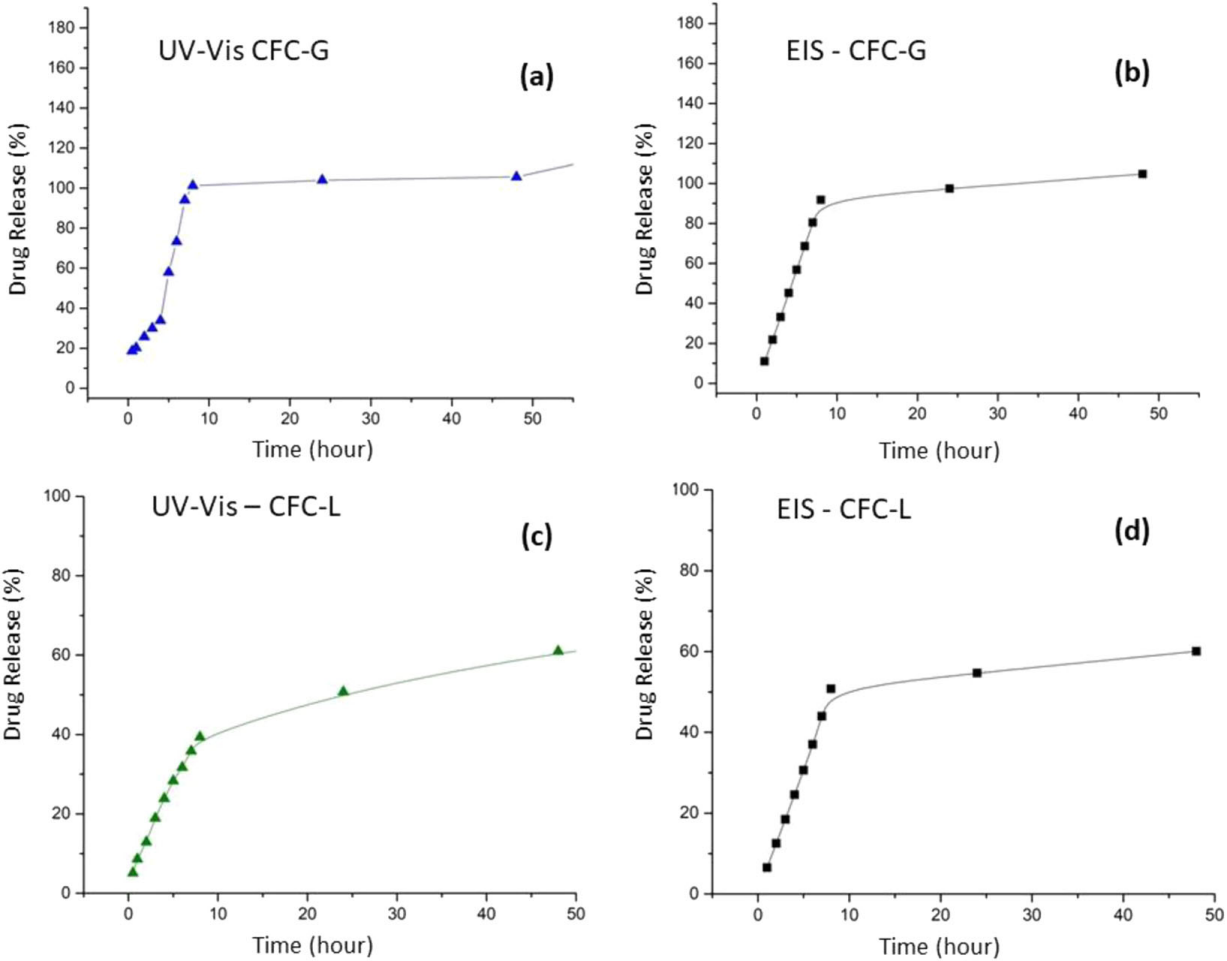
Release profiles obtained by **a**, **c** UV–Vis and **b**, **d** EIS

Together with this cumulative sum assumption, correction factors need to be applied to both the CFC-L and CFC-G samples. The values applied were 12 (CFC-L) and 74 (CFC-G). These factors were chosen based on the percentages of release found in the results obtained by UV–Vis. Correction factors were obtained by comparing the release profiles obtained by UV–Vis and EIS. When these two profiles are divided (values obtained by EIS divided by values obtained by UV–Vis) the results obtained from this ratio were 12 for samples of CFC-L, and 74 for samples of CFC-G. Figure 17 shows the profile for samples of CFC-G (Fig. 17a, b) and CFC-L (Fig. 17c, d).

The EIS results are more accurate than analysis using UV–Vis, which is limited by its sensitivity linked to each solution’s absorbance characteristics. The UV–Vis has a limit on the absorbance intensity such that solutions with low concentration and low absorbance cannot be analyzed by this technique. Using EIS, the responses of the applied potential can be observed, even if the values are low.

Using the HPLC the analysis good sensitivity on the measurements will be obtained but the solution must be diluted exactly because this high sensitivity, obligating a sample preparation. The samples used here do not have a dilution step when analyzed by EIS, and the sample did not need to be discarded after the measurement, as happens when the HPLC technique is used.

## Conclusion

Measurement of drug concentration in solution by EIS is shown to be a promising technique by providing drug in solution presence quantification by varying the system’s electrochemical behavior. With this, it is possible to carry out measurements without the need of the aliquot removal, as the conventional methods require, with in situ monitoring of the drug release process in solution, without sample degradation and with high precision in the results, using the relation of the cumulative quantity of the drug under consideration, and analyzing the specified corrective factor for impedance to obtain the conventional release profile, as is commonly observed during UV–Vis measurements.

The drug release measurement standardization by EIS was performed in this work in order to maximize the noise reduction related to external interferences in the performed measurements. The BIAS variation and the CV measurements enabled the determination of the linearity region which provides EIS measurements with DC potential of smaller possible values, guaranteeing a good current response.

The results showed that the molecular structure of the studied drugs interferes in the release process by increasing, or decreasing, the resistance values according to the size of the chain.
